# Sexual function assessment in men with PTSD

**DOI:** 10.1192/j.eurpsy.2021.1470

**Published:** 2021-08-13

**Authors:** R. Maalej, G. Hamdi, D. Felfel, H. Ben Ammar, A. Maamri, Y. El Kissi, H. Zalila

**Affiliations:** 1 Psychiatry Department, Razi, manouba, Tunisia; 2 Psychiatry, Tunisian Society of Clinical Sexology, TUNIS, Tunisia

**Keywords:** SEXUAL FUNCTION, ptsd

## Abstract

**Introduction:**

Exposure to extreme traumatic events can lead to post-traumatic stress disorder (PTSD). This disorder affects emotional, social and professional functioning. Recent studies suggest that it can lead to sexual dysfunction.

**Objectives:**

The aim of this study is to compare the level of sexual dysfunction between men with PTSD and control subjects.

**Methods:**

A total of 30 male PTSD patients and 30 controls were included in this study. We used the Post-Traumatic Stress Disorder Check Scale (PCLS) to assess the intensity of PTSD symptoms and the International Erectile Function Index (IIEF15) to assess sexual dysfunction of both patients and controls.

**Results:**

The mean IIEF-15 score was 51.16 ± 6.82 in patients followed for PTSD versus 77.33 ± 2.02 in healthy controls with a non-significant difference (p = 0.26). Three patients (10%) had an alteration of desire while the control reported only dysfunction but there was no significant difference between the mean scores of IIEF-SD (p = 0.22). No patient or control had erectile dysfunction and there was no significant difference between the IIEF-EF sub-scores in the 2 groups (p = 0.20). The mean sexual intercourse satisfaction (SD) score in the patients was 5.13 ± 1.10 versus 8.86 ± 0.40 with a non-significant difference (p = 0.09). Altered satisfaction with intercourse was noted in 15% (n = 5) of subjects with PTSD versus a single control.

**Conclusions:**

It is important that practitioners address the subject of sexuality in patients followed for PTSD and refer their patients, if necessary, to a sexology consultation.

